# Psychosocial Correlates of Adherence to Mind–Body Interventions

**DOI:** 10.1007/s11121-025-01810-1

**Published:** 2025-05-06

**Authors:** Elizabeth Jean Duraney, Madhura Phansikar, Ruchika Shaurya Prakash

**Affiliations:** 1 Department of Psychology, The Ohio State University, 1835 Neil Ave Columbus, Columbus, OH 43210 USA; 2 Center for Cognitive and Behavioral Brain Imaging, The Ohio State University, Columbus, OH 43210 USA

**Keywords:** Mind–body interventions, Intervention adherence, Cognitive functioning, Depression, Emotion dysregulation

## Abstract

Mind–body interventions involve practices that intentionally combine mental and physical fitness, showing promise for improving psychological and cognitive health in older adults. Limited research exists on adherence to these interventions and the demographic and psychosocial factors that may predict variability in compliance. In the current study, we identified key correlates—demographic, psychosocial, and cognitive—of adherence to two mind–body interventions. Baseline and intervention data were analyzed together from a randomized controlled trial of older adults who participated in two four-week mind–body interventions and completed practice logs (*n* = 60). Adherence was defined as the average weekly self-reported minutes of homework practice during the intervention. Baseline correlates included education, sex assigned at birth, working memory score, emotion dysregulation, positive and negative affect, trait mindfulness, and depression. Partial least squares regression was used to identify latent components. A significant one-component solution from the final model explained 23.08% of the variance in practice minutes. Greater adherence was associated with mild depressive symptoms, difficulties with emotion regulation, and lower working memory scores. These findings suggest that participants with mild emotional and cognitive difficulties may be more likely to adhere to mind–body interventions. These results emphasize the target population likely to engage in mind–body interventions and may be valuable for designing tailored interventions and developing strategies to maximize adherence. This study was registered on ClinicalTrials.gov (#NCT03432754) on February 14, 2018.

Older adults represent one of the fastest-growing segments of the population, with the number of individuals aged 65 and older projected to nearly double to 95 million by 2060 (Vespa et al., 2018). Aging is associated with well-documented declines in higher-order cognitive functions, including information processing, episodic memory, executive functioning, and working memory (Park & Reuter-Loernz, 2009; Salthouse, 2010), as well as shifts in patterns of emotion regulation strategy use (Whitmoyer et al., 2023; Allen & Windsor, 2017). These shifts in cognitive and affective processing have negative downstream effects on quality of life (Baernholdt et al., 2012). To mitigate the adverse effects of age-related changes, there is a growing focus on understanding the efficacy of mind–body interventions for older adults.

Mind–body interventions, as defined by the National Center for Complementary and Integrative Health, encompass practices that intentionally combine mental and physical fitness (NCCIH, 2017). These interventions include various approaches such as mindfulness meditation (Reangsing et al., 2021) and lifestyle education programs (Fourteau et al., 2020; Ngandu et al., 2022; Wu et al., 2019). The effects of these interventions on metrics of cognitive and emotional functioning in older adults have been mixed. While some studies support the positive effects of these interventions (Reangsing et al., 2021; Wang et al., 2020; Mirabito & Verhaeghen, 2023; Fourteau et al., 2020), others report null effects (Sanchez-Lara et al., 2022; Han et al., 2022; Samimy et al., 2022). One potential explanation for these discrepant findings across studies could be variation in adherence to the prescribed interventions.

The World Health Organization (WHO; 2003) defines adherence as “the extent to which a person’s behavior agrees with the recommendations of a health care provider.” Despite growing interest in evaluating the efficacy of mind–body interventions on various outcomes for older adults, few studies provide detailed adherence data. A recent scoping review of adherence to mindfulness-based interventions for women with cancer found that only 70% of the studies reported any adherence data (Stanic et al., 2021). Similarly, a systematic review examining the quality of studies assessing the effects of exercise interventions found that adherence was among the most underreported metrics (Ibeggazene et al., 2022). Furthermore, empirical evidence suggests that only two-thirds of studies provide a definition of adherence (Winter et al., 2022).

Further complicating adherence to mind–body interventions is the heterogenous operationalization of adherence across studies. While some studies define adherence as completing 50% of an intervention, others require 100% completion of the program to be considered adherent (Winter et al., 2022). A recent review of adherence to home practice in mindfulness-based interventions found significant variability in adherence, defined as the percentage of recommended homework minutes completed. Some studies reported adherence rates as low as 31%, while others reported rates over 100%, indicating participants practiced more than the recommended amount (Baydoun et al., 2021). Similarly, rates of adherence to lifestyle education interventions range from as low as 5% (de Souto Barreto et al., 2021) to as high as 95% (Coley et al., 2019). Understanding adherence to mind–body interventions is crucial as low adherence undermines the validity of treatment effects. Given the variability in adherence across studies, it is essential to identify factors influencing adherence and leverage these findings to design interventions that promote compliance, particularly for those at risk of low adherence.

The existing literature on correlates of adherence to mind–body interventions for older adults is limited with studies identifying a broad array of potential predictors. Among these, depression has received the most support as a key correlate of adherence in both mindfulness-based interventions and lifestyle education interventions (Baydoun et al., 2021; Canby et al., 2021; Coley et al., 2019; Hearn & Finlay, 2018; Picorelli et al., 2014). However, the direction of this relationship is inconclusive. Although many studies demonstrate a negative relationship between symptoms of depression and adherence (Tamagawa et al., 2015; Barrett et al., 2019; Picorelli et al., 2014; Coley et al., 2019), a few studies report a positive relationship (Canby et al., 2021; Siebenhüner et al., 2021). These discrepant findings may be attributed to differences in assessment measures and variability in the range of depressive symptoms across studies. Further research is needed to clarify the role of depressive symptoms in predicting adherence.

The existing literature also provides preliminary support for personality characteristics (Canby et al., 2021; Forbes et al., 2018) and anxiety (Baydoun et al., 2021; Gutierrez et al., 2020) as predictors of adherence to mindfulness-based interventions. Additionally, in older adults, there is empirical support associating female sex (Lam et al., 2015), higher education (Coley et al., 2019), higher socioeconomic status (Picorelli et al., 2014), and preserved cognitive functioning (Picorelli et al., 2014) with increased adherence to lifestyle education interventions. Overall, given the limited research, there is a lack of clarity on the key correlates of adherence to mind–body interventions, highlighting the need for further research.

Identifying correlates of adherence to mind–body interventions serves several critical functions. It helps determine whether differences in intervention efficacy stem from the intervention itself or from a lack of adherence, helping identify strategies to enhance adherence in future studies. Although the existing literature has begun to examine correlates of adherence, studies have primarily focused on individual correlates (e.g., Forbes et al., 2018; Gutierrez et al., 2020). However, examining correlates in isolation is inadequate. By considering only a few correlates of adherence at a time, existent research fails to capture the combined contributions of demographic, cognitive, and psychosocial factors. To address this gap, the current project utilized partial least squares regression (PLSR), a statistical method that enables the combined and individual examination of multiple predictor variables while correcting for collinearity among these variables. Furthermore, PLSR allows for the identification of latent components spanning a multitude of relevant factors, that collectively predict adherence. 

The aim of the present study was to identify baseline correlates of adherence to two mind–body interventions: a mindfulness-based attention training (MBAT) program and a psychoeducation-based, lifestyle education training (LifeEd) program. Given the high attendance to the in-person sessions (90%), adherence was operationalized as the average minutes of homework practice completed by the participants over the four weeks of the intervention. We hypothesized that the following demographic, psychosocial, and cognitive variables would be related to intervention adherence: age (Coley et al., 2019), education (Coley et al., 2019; Geurts et al., 2021), sex (Lam et al., 2015; Mascaro et al., 2020), cognitive functioning (Morris et al., 2021; Picorelli et al., 2014), metrics of emotion dysregulation (Dorandish & Abouzari, 2022), mindfulness (Forbes et al., 2018), positive and negative affect (Barrett et al., 2019), and depression (Canby et al., 2021; Barrett et al., 2019; Tamagawa et al., 2015). A PLSR model was constructed using cross-validation and variable loadings were interpreted to assess relationships between demographic, psychosocial, and cognitive variables with intervention adherence.

## Methods

### Study Design and Sample

The parent study was a four-week parallel, single-blind randomized controlled trial investigating the preliminary effects of two mind–body interventions: a mindfulness-based attention training intervention (MBAT), which served as the experimental group, and compared it to an active control group, the lifestyle education intervention (LifeEd) group (ClinicalTrials.gov #NCT03432754). The intervention was conducted between November 2014 and March 2015 and the recruitment started shortly before in 2014. Data and code for the present analyses can be found on Open Science Framework. See Whitmoyer et al., (2020) and Samimy et al., (2022) for additional details. Participants aged 60 to 74 years, without prior exposure to mindfulness training, meditation, and yoga, and without self-reported neurological, psychiatric, or inflammatory disorders, were considered. Individuals were eligible for the study if they were cognitively normal, with a Mini-Mental Status Examination score of > 23 (Folstein et al., 1975), and with a score of ≤ 10 on the Geriatric Depression Scale (GDS; Yesavage et al., 1982).

Sample size was determined a priori for the parent trial based on prior research (Mrazek et al., 2013). We screened 147 participants and initially randomized 75 participants into the study. However, one potential participant who had initially indicated interest in the study was randomized in error. No data was collected from this individual. Thus, a total of 74 participants (*n* = 37 per group) were eligible and randomized into the study. Of these 74 individuals, fourteen participants did not have intervention adherence data, resulting in a final sample size of 60 participants for this analysis (LifeEd *n* = 34; MBAT *n* = 26). For the power analysis, and CONSORT diagram, see Whitmoyer et al., 2020. The study was approved by the Institutional Review Board of The Ohio State University and participants provided informed consent.

### Study Procedures

Data was collected in the Clinical Neuroscience Laboratory at The Ohio State University. Upon meeting the eligibility criteria, participants underwent a 2.5 h, in-person session comprising questionnaires and cognitive assessments. Following the assessment, participants were randomly assigned to one of two mind–body intervention groups: MBAT or LifeEd. The control group was an attention-matched control group. We selected the LifeEd intervention to provide meaningful and beneficial information for participants while matching the intervention in all aspects except for the mindfulness component. Both interventions occurred once a week for 1.5 h for four weeks. Participants were asked to engage in homework practices for 40 min on the remaining six days each week throughout the four weeks.

The MBAT intervention was adapted from the traditional mindfulness-based stress reduction (MBSR) protocol (Kabat-Zinn, 1982). Group sessions, led by an instructor trained in—MBSR, included a didactic component focused on mindfulness, meditation practices, such as breathing exercises body scans, and homework review. The group-based LifeEd intervention comprised of lectures on scientific health and lifestyle information led by an exercise physiologist. Classes included a didactic component based on concepts from “*The Culprit and the Cure: Why Lifestyle is the Culprit Behind America’s Poor Health and how Transforming That Lifestyle can be the Cure*” (Aldana, 2005), along with low-intensity stretching and toning exercises, and homework review. Both groups were matched on intervention duration, recommended practice minutes, format of delivery, and experimenter contact time.

## Measures

### Demographic

Participants self-reported their current age in years, assigned sex at birth, and the number of years of education at the time of enrollment in the study.

### Psychosocial

#### Geriatric Depression Scale (GDS)

The GDS (Yesavage et al., 1982) is a 30-item scale designed to assess symptoms of depression in the past week. Participants respond “yes” or “no” to each item. A total score is calculated by summing the number of “yes” responses. Participants with a score of ≤ 10 were included. Cronbach’s alpha at baseline was 0.69.

#### Positive and Negative Affect Schedule- Short Form (PANAS-SF)

The PANAS-SF (Thompson, 2007) is a 10-item scale assessing state levels of positive and negative affect. Items represent an emotional state, participants rate how much they are currently experiencing each emotion. Responses range from 1 (very slightly or not at all) to 5 (extremely). A total score is calculated for each subscale by summing responses after reverse coding. Higher scores for each subscale indicate higher positive or negative affect. Cronbach’s alpha at baseline was 0.80.

#### Mindfulness Attention Awareness Scale (MAAS)

The MAAS (Brown & Ryan, 2003) is a 15-item scale designed to assess dispositional mindfulness. The items assess participants’ general level of present-moment awareness in everyday situations. Participants rate each item on a Likert scale ranging from 1 (almost always) to 6 (almost never). A total score is calculated as an average of the individual item responses, where a higher score indicates a higher level of dispositional mindfulness. Cronbach’s alpha at baseline was 0.77.

#### Difficulties in Emotion Regulation Scale (DERS)

The DERS (Gratz & Roemer, 2004) is a 36-item scale designed to assess emotion dysregulation. Participants indicate how often each item applies to them, with responses ranging from 1 (almost never) to 5 (almost always). The DERS consists of five subscales. **1) Non-acceptance of emotional responses (Non-acceptance)** measures non-accepting or negative reactions to emotional distress. An example item is “When I’m upset, I become angry with myself for feeling that way.” Cronbach’s alpha = 0.87. **2) Difficulty engaging in goal-directed behavior (Goals)** measures difficulty pursuing one’s goals while experiencing emotional distress. An example item is “When I’m upset, I have difficulty getting work done.” Cronbach’s alpha = 0.88. **3) Impulse control difficulties (Impulse)** measures difficulties controlling impulsive reactions. An example item is “I experience my emotions as overwhelming and out of control.” Cronbach’s alpha = 0.70. **4) Lack of emotional awareness (Awareness)** measures difficulties in being aware of one’s emotions. An example item is “I pay attention to how I feel.” Cronbach’s alpha = 0.82. **5) Limited access to emotion regulation strategies (Strategies**) measures the belief that one cannot regulate their emotions. An example item is “When I’m upset, I believe that I will remain that way for a long time.” Cronbach’s alpha = 0.74. **6) Lack of emotional clarity (Clarity**) measures the extent to which one understands their emotions. An example item is “I have no idea how I am feeling.” Cronbach’s alpha = 0.71.

### Cognitive Functioning

#### Working Memory Index (WMI)

Working memory is the capacity to retain and manipulate information in short-term memory (Baddeley, 1992). The WMI was derived from the Wechsler Adult Intelligence Scale-IV subtests of Digit Span and Arithmetic (Wechsler, 2008). The WMI was calculated as an overall age-normed standardized score based on the individual scores on the two subtests. A higher score on the WMI indicates better working memory.

### Practice Minutes

Practice minutes, calculated as the average minutes of homework practiced each week, was the outcome variable. This was measured using tracking logs completed by participants. Participants were asked to fill out the log after their homework practice, recording the day, the type of homework practice, and the start and end times for each practice.

### Statistical analysis

Of the 74 participants randomized into the study, fourteen participants did not submit tracking logs and had no intervention adherence data. Thus, the analysis for the current study included 60 participants who provided adherence data. All data were winsorized, capping individual scores ± 2.5 *SDs* from the mean with the corresponding value at 2.5 *SD* for that variable. Less than 5% of the total number of values were outlier corrected. Analyses were conducted in R (R Core Team, 2000) using the *pls* (Liland et al., 2022) and *plsVarSel* (Mehmood et al., 2012) packages*.* We analyzed the data using PLSR, a dimension reduction technique that optimizes the covariance between predictor and outcome variables. PLSR yields latent components that are a linear combination of predictor variables. We built a PLSR model with practice minutes as the outcome variable and the following 14 variables as predictors: age, sex, education, depression, mindfulness, positive affect, negative affect, working memory, and the six subscales of the DERS.

For the first PLSR model, we used the *kernelpls* algorithm and leave-one-out cross-validation (LOOCV) with all predictor variables to determine the optimal number of components which explained the most variance in the outcome while avoiding overfitting. This number was selected based on the lowest root mean squared error of prediction (RMSEP) and the absolute minimum predicted residual error sum of squares (PRESS) statistic. Next, we examined the variable importance in projection (VIP) statistic, a weighted sum of squares of the component weights, to identify the variables that were not contributing to the model. Variables with a VIP value below 0.80 were removed from the model (Sawatsky et al., 2015). We constructed a refined PLSR model by specifying the optimal number of components and the reduced set of variables derived from the initial PLSR. The significance of this pruned model was assessed through a linear regression model, where the predictors consisted of the PLSR component scores. We interpreted the magnitude and direction of the variables loading onto that component ( >|.17|; Ng et al., 2021) to determine the relationship between the variables and adherence.

## Results

Participants had a mean age of 67 years and an average education duration of 17 years. Fifty-seven percent of our participants were female. Demographic and baseline characteristics are presented in Table 1. The average minutes of homework practice each week were 247.2 min (*SD* = 134.2), compared to the recommended weekly practice time of 240 min. Practice minutes ranged from 21.25 min to 621.8 min, on average, each week.

**Table 1:** Demographic and baseline characteristics of the sample (n = 60)

Measure	Mean or N	SD	Range
Age (yrs.)	66.7	3.95	60–74
Sex, female (*n*)	34		
Education (yrs.)	16.8	2.6	12–24
Race/Ethnicity			
White or Caucasian	51		
Black or African American	7		
Hispanic or Latino	1		
Not reported	1		
MAAS	4.4	0.6	3–5.7
PANAS-PA	13.8	4.5	7–23
PANAS-NA	5.8	1.1	5–9.5
GDS	3.7	2.9	0–10
DERS	62.6	12.8	41–94.9
WMI	106	12.8	73–139.1
Practice Minutes	247.2	134.2	21.3–621.8

*a. Note.* MAAS = Mindful Attention Awareness Scale; PANAS-PA = Positive and Negative Affect Schedule – Positive Affect; PANAS-NA = Positive and Negative Affect Schedule – Negative Affect; GDS = Geriatric Depression Scale; DERS = Difficulties in Emotion Regulation Scale; WMI = Working Memory Index; Practice Minutes = Average weekly minutes of homework practiced

**Fig. 1:**
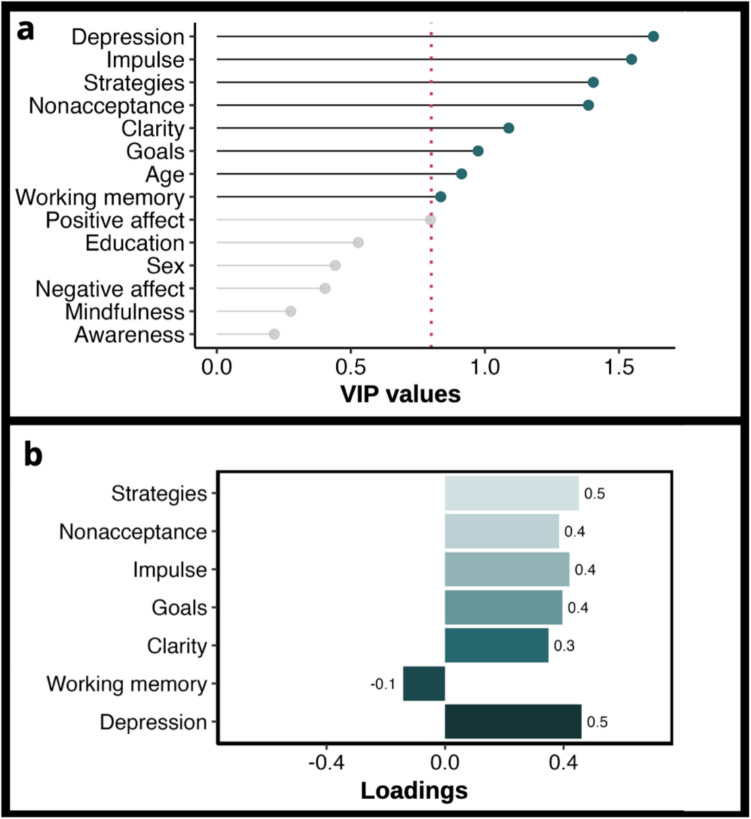
**A** Variable Importance in Projection (VIP) Values and *b*. Loading Plot for PLSR Model. *Note*. **a**) The variables having a VIP value > .80 (indicated by green highlighted circles) were considered to contribute strongly to the model and were included in the pruned PLSR model. **b**) Only variables with a loading value >|.17| were considered to contribute significantly to the model and are included in this plot

We conducted a PLSR with 14 predictors and total practice minutes as the outcome variable. The initial PLSR model showed that a one-component solution was the best fit. It had the lowest RMSEP value (125.7). This one component explained 25.5% of the variance in practice minutes. We then examined the VIP values of all predictors (presented in Fig. 1A), specifying a one-component solution, to identify the correlates that contributed strongly to the model. The variables with a VIP value greater than 0.8, representing variables making a substantial contribution to the model, were age, depression, the WMI, and five of the subscales from the DERS (clarity, goals, impulse, nonacceptance, and strategies). We then constructed a pruned PLSR model that included eight of the 14 variables with VIP > 0.8 and specified a one-component solution. The RMSEP of this model was comparable with the earlier model (124.3) and explained 23.08% of the variance in practice minutes.

The significance of this pruned model was tested by conducting a linear regression with the component scores as the predictor variables and practice minutes as the outcome variable. The model was significant (*β* = 35.92*, p* < 0.001), suggesting that the component scores were significantly associated with practice minutes. As the model was significant, we examined the loadings of the variables to determine the association between individual variables and practice minutes. Variables with significant positive loadings ( >|.17|) (presented in Fig. 1B) were positively associated with practice minutes. These included depression (0.46), strategies (0.45), impulse (0.42), goals (0.40), nonacceptance (0.39), and clarity (0.35). Working memory had a negative loading (−0.14), indicating a negative association with practice minutes.

The results suggest that higher scores on the depression scale and emotion dysregulation subscales and lower working memory were associated with increased engagement. We examined the mean and range of these variables to better characterize our study sample. Our sample had an average depression score of 3.7 (range = 0–10), indicating the absence of clinical depression. The average scores and ranges for the emotion dysregulation subscales were: clarity = 8.7 (5–15), goals = 10.6 (5–20), impulse = 8.3 (6–16), nonacceptance = 9.8 (6–19), and strategies = 11.2 (8–19). All these scores fall below the means of the validation sample, which included patients at an outpatient psychology clinic (Hallion et al., 2018). This suggests overall lower levels of emotion dysregulation in the study sample. The mean and range for WMI score was 106 (73–139.1). Compared to normative WAIS-IV (Wechsler, 2008) scores, this score indicates average levels of working memory in our sample. Overall, our sample reported lower levels of depression and emotion dysregulation and normative levels of working memory. The results of our pruned regression model are therefore discussed in the context of these average scores.

## Discussion

The aim of this study was to identify latent component(s) comprising demographic, psychosocial, and cognitive variables associated with adherence to two mind–body interventions among healthy older adults. Our findings indicate that a latent component consisting of mild depressive symptoms, facets of emotion dysregulation, and working memory was a key correlate of adherence—reflecting greater engagement with homework practices—in both interventions. Specifically, higher levels of mild depressive symptoms, greater emotion regulation difficulties, and lower working memory were associated with greater adherence to homework practices.

In our sample of older adults, we observed a positive association between higher levels of depressive symptoms and increased intervention adherence. Previous research suggests that the relationship between depressive symptoms and adherence is complex, and at times, contradictory. While few studies suggest that higher depression is associated with greater engagement (Canby et al., 2021; Siebenhüner et al., 2021), most evidence suggests an inverse relationship between higher depressive symptoms and lower adherence in both mindfulness and lifestyle education interventions (Tamagawa et al., 2015; Barrett et al., 2019; Picorelli et al., 2014; Coley et al., 2019). For example, a randomized controlled trial comparing mindfulness meditation and exercise interventions found that fewer depressive symptoms were associated with greater adherence for both interventions (Barrett et al., 2019).

Although these findings may seem contradictory to the present results that suggest a positive association between depression scores and adherence rates, it is important to consider the range of depressive symptoms in our sample, which was subthreshold. Based on our inclusion criteria for the parent trial, participants scoring above 10 on the GDS were excluded from the study, resulting in a mean depression score of 3.7 (range = 0–10). Our findings suggest that individuals approaching the upper end of this depression range (a score of 10) showed greater intervention adherence, as indicated by higher engagement in homework practices. Given that our sample of participants did not include those with elevated levels of depressive symptoms, the positive relationship we identified, may still align with other studies that demonstrate lower levels of depression to be associated with greater intervention adherence. In the extant literature at least one study identified a positive relationship between depressive symptoms and adherence. Specifically, in an examination of predictors of out-of-class mindfulness practice, greater depressive symptoms, assessed using the Inventory of Depressive Symptomatology (IDS; Rush et al., 1986), predicted greater intervention adherence during the intervention and at post-intervention (Canby et al., 2021). The average depressive symptoms were 23.01, indicating mild depression, aligning with our findings (Canby et al., 2021). Research in samples with a range of depression symptoms is needed to fully elucidate this relationship.

Higher scores on several dimensions of emotion dysregulation—including the strategies, goals, impulse, nonacceptance, and clarity subscales of the DERS—were associated with greater intervention adherence. The only subscale unrelated to adherence was the awareness subscale, which measures the ability to recognize and attend to emotions. This subscale has been shown to inversely correlate with measures of mindfulness (Goodall et al., 2012; Vujanovic et al., 2010), and notably, neither this subscale nor trait mindfulness correlated with adherence in our study. The extant literature on trait mindfulness as a correlate of adherence to mind–body interventions is limited and inconclusive. While one study supports its predictive value (Forbes et al., 2018), others do not (Castro et al., 2021; Barrett et al., 2019). Supporting our results, a methodologically similar study examining correlates of mindfulness meditation and exercise practice found mindfulness correlated with adherence to the exercise intervention but not the mindfulness intervention (Barrett et al., 2019). This highlights the need for further research to clarify the relationship between dispositional mindfulness and adherence to mind–body interventions.

To our knowledge, no prior studies have examined emotion dysregulation as a correlate of adherence to mind–body interventions. In the broader literature, lower levels of emotion dysregulation are associated with greater adherence to medication protocols (Dorandish & Abouzari, 2022). Given that emotion dysregulation has strong theoretical ties to depression (Bradley et al., 2011; Joormann & Stanton, 2016), it may similarly influence adherence to mind–body interventions. It is important for future studies to examine this correlate of adherence further.

Finally, we found that older adults with lower working memory scores were more adherent to the mind–body interventions, completing more of the prescribed practices. Older adults with lower working memory may have had more opportunity to observe functional changes resulting from the intervention, potentially increasing their motivation to engage. Alternatively, these findings may suggest mind–body interventions are accessible even to those with lower working memory scores which in turn leads to better adherence. The literature on metrics of cognitive functioning as correlates of adherence to mind–body interventions is limited. However, there is a growing body of literature examining the impact of cognitive functioning in predicting adherence to other behavioral interventions. Systematic reviews of physical activity interventions (Morris et al., 2021; Picorelli et al., 2014) and cognitive training interventions (He et al., 2022; Turunen et al., 2019) evince support for better cognitive abilities, such as memory, executive functioning, and processing speed, to be associated with higher adherence among older adults. In contrast to our findings, the existing literature suggests a positive relationship between cognitive abilities and intervention adherence, albeit, among physical activity and cognitive-training interventions. There is prior evidence that different baseline factors predicted adherence to a mindfulness intervention as compared to an exercise intervention (Barrett et al., 2019), suggesting unique factors may influence adherence to these different practices.

The results of the current study should be considered in the context of several limitations. The inclusion criteria for the parent trial restricted our sample to participants with GDS scores ≤ 10 (Yesavage et al., 1982). Future research should examine correlates of adherence in a sample of older adults with a broader range of depressive symptoms. Additionally, recent meta-analyses have shown that shorter forms of the GDS (GDS-15 and 10) have higher accuracy and diagnostic performance than GDS-30 (Krishnamoorthy et al., 2020). We recommend future studies implement the short versions. Our results are further constrained by the homogeneity of the sample. Participants in our sample were predominantly non-Hispanic and white (85.9%), which has implications for the generalizability of our study results. Recent research has shown that racial and ethnic differences in emotion regulation strategies exist in more than 50% of the studies (Weiss et al., 2022). A recent well-powered study (*n*= 1614) examined cognitive functioning among different racial groups in the context of social determinants of health—environments that we live and work in which influence our well-being. Minoritized populations residing in disadvantaged neighborhoods were reported to have poorer cognitive functioning (Wong et al., 2023). Given these racial disparities in correlates of adherence, it would be important to examine emotional and cognitive health in an inclusive, diverse sample to improve reliability and generalizability of findings. This can be achieved by using an evidenced-based, multi-pronged recruitment approach starting with cultural competency training for staff members, and using recruitment methods such as establishing community connections, using culturally-sensitive recruitment materials, and using online platforms popular among minoritized populations (Bodicoat et al., 2021).

Finally, our results are limited by the use of self-report logs for tracking intervention adherence. While adherence to home practice minutes is most often recorded using self-report diaries (Baydoun et al., 2021; Parsons et al., 2017), recent research suggests these measures may overestimate adherence (Flett et al., 2019). These findings should be replicated in a dataset with both subjective (e.g., self-report logs) and objective measures (e.g., mobile-based tracking). Future research should examine factors associated with adherence in a broad way by using a mixed-methods approach that combines measures of adherence with qualitative reports on barriers and facilitators of adherence. This allows for the incorporation of social determinants of health, such as social support, health literacy, healthcare access, and racial and ethnic identity, to help develop a model of intersecting variables that may contribute to adherence rates.

The present study contributes to the growing body of literature on mind–body interventions as tools for promoting cognitive and emotional functioning in older adults. We identified a set of predictors—mild depressive symptoms, emotion dysregulation, and lower working memory scores—associated with greater adherence to two mind–body interventions. Given that there are currently no available pharmacological interventions to reverse age-related declines in cognitive functioning, behavioral interventions, especially those facilitating mind–body connections, are increasingly being examined for their preventative potential in delaying or slowing cognitive decline with advancing age. Our findings suggest that individuals with mild depression, emotion dysregulation, and lower working memory can adhere to these brief, four-week, mind–body interventions. Although these results need to be replicated in future large-scale trials with a more representative sample, data from this pilot study evinces support for the use of these preventative interventions with older adults beginning to show declines in these cognitive and affective domains. Furthermore, understanding correlates of adherence to mind–body interventions is critical to successful dissemination and implementation of these interventions—given low levels of adherence are likely to be associated with worse outcomes. Thus, future research should also explore ways to promote adherence. Based on our findings, considerations such as motivational interviewing may be warranted for individuals at risk of low adherence.

## Data Availability

Data and code for the present analyses can be found on Open Science Framework.

## References

[CR1] Aldana, S. (2005). *The culprit and the cure: Why lifestyle is the culprit behind America’s poor health*. Maple Mountain Press.

[CR2] Allen, V. C., & Windsor, T. D. (2017). Age differences in the use of emotion regulation strategies derived from the process model of emotion regulation: a systematic review. *Aging & Mental Health*, *0*(0), 1–14. 10.1080/13607863.2017.1396575 10.1080/13607863.2017.1396575 29148830

[CR3] Baddeley, A. (1992). Working memory. *Science,**255*(5044), 556–559. 10.1126/science.1736359 1736359 10.1126/science.1736359

[CR4] Baernholdt, M., Hinton, I., Yan, G., Rose, K., & Mattos, M. (2012). Factors associated with quality of life in older adults in the United States. *Quality of Life Research,**21*, 527–534. 21706127 10.1007/s11136-011-9954-z PMC3593634

[CR5] Barrett, B., Torres, E. R., Meyer, J., Barnet, J. H., & Brown, R. (2019). Predictors of mindfulness meditation and exercise practice, from MEPARI-2, a randomized controlled trial. *Mindfulness,**10*, 1842–1854. 10.1007/s11136-011-9954-z 31938076 10.1007/s12671-019-01137-3 PMC6959135

[CR6] Baydoun, M., Moran, C., McLennan, A., Piedalue, K. A. L., Oberoi, D., & Carlson, L. E. (2021). Mindfulness-based interventions in cancer survivors: a systematic review of participants’ adherence to home practice. *Patient preference and adherence*, 1225–1242. 10.2147/PPA.S267064 10.2147/PPA.S267064 PMC8200136 34135575

[CR7] Bodicoat, D. H., Routen, A. C., Willis, A., Ekezie, W., Gillies, C., Lawson, C., ... & Khunti, K. (2021). Promoting inclusion in clinical trials—a rapid review of the literature and recommendations for action. *Trials*, *22*, 1–11. 10.1186/s13063-021-05849-7 10.1186/s13063-021-05849-7 PMC8643184 34863265

[CR8] Boutron, I., Altman, D. G., Moher, D., Schulz, K. F., Ravaud, P., & CONSORT NPT Group*. (2017). CONSORT statement for randomized trials of nonpharmacologic treatments: a 2017 update and a CONSORT extension for nonpharmacologic trial abstracts. *Annals of internal medicine*, *167*(1), 40-47. 10.7326/M17-0046 10.7326/M17-0046 28630973

[CR9] Bradley, B., DeFife, J. A., Guarnaccia, C., Phifer, J., Fani, N., Ressler, K. J., & Westen, D. (2011). Emotion dysregulation and negative affect: Association with psychiatric symptoms. *The Journal of Clinical Psychiatry,**72*(5), 6427. 10.4088/JCP.10m06409blu 10.4088/JCP.10m06409blu PMC4605672 21658350

[CR10] Brown, K. W., & Ryan, R. M. (2003). The benefits of being present: Mindfulness and its role in psychological well-being. *Journal of Personality and Social Psychology,**84*(4), 822. 10.1037/0022-3514.84.4.822 12703651 10.1037/0022-3514.84.4.822

[CR11] Canby, N. K., Eichel, K., Peters, S. I., Rahrig, H., & Britton, W. B. (2021). Predictors of out-of-class mindfulness practice adherence during and after a mindfulness-based intervention. *Psychosomatic Medicine,**83*(6), 655–664. 10.1097/PSY.0000000000000873 33038188 10.1097/PSY.0000000000000873 PMC8024418

[CR12] Castro, A., García-Palacios, A., López-Del-Hoyo, Y., Mayoral, F., Pérez-Ara, M. Á., Baños, R. M., ... & Gili, M. (2021). Predictors of adherence in three low-intensity intervention programs applied by ICTs for depression in primary care. *International journal of environmental research and public health*, *18*(4), 1774. 10.3390/ijerph18041774 10.3390/ijerph18041774 PMC7918657 33670353

[CR13] Coley, N., Ngandu, T., Lehtisalo, J., Soininen, H., Vellas, B., Richard, E., ... & Perret, B. (2019). Adherence to multidomain interventions for dementia prevention: Data from the FINGER and MAPT trials. *Alzheimer's & Dementia*, *15*(6), 729–741. 10.1016/j.jalz.2019.03.005 10.1016/j.jalz.2019.03.005 31047857

[CR14] de Paula, J. J., Diniz, B. S., Bicalho, M. A., Albuquerque, M. R., Nicolato, R., de Moraes, E. N., ... & Malloy-Diniz, L. F. (2015). Specific cognitive functions and depressive symptoms as predictors of activities of daily living in older adults with heterogeneous cognitive backgrounds. *Frontiers in Aging Neuroscience*, *7*, 139. 10.3389/fnagi.2015.00139 10.3389/fnagi.2015.00139 PMC4507055 26257644

[CR15] de Souto Barreto, P., Pothier, K., Soriano, G., Lussier, M., Bherer, L., Guyonnet, S., ... & Vellas, B. (2021). A web-based multidomain lifestyle intervention for older adults: the eMIND randomized controlled trial. *The Journal of prevention of Alzheimer's disease*, *8*, 142–150. 10.14283/jpad.2020.70 10.14283/jpad.2020.70 PMC7754697 33569560

[CR16] Dorandish, F., & Abouzari, F. (2022). Investigating the role of difficulty in emotion regulation, Distress Tolerance and perceived stress in predicting treatment adherence in patients with type 2 diabetes. *Quarterly Journal of Nursing Management,**11*(3), 38–48.

[CR17] Flett, J. A., Fletcher, B. D., Riordan, B. C., Patterson, T., Hayne, H., & Conner, T. S. (2019). The peril of self-reported adherence in digital interventions: A brief example. *Internet Interventions,**18*, 100267. 10.1016/j.invent.2019.100267 31890620 10.1016/j.invent.2019.100267 PMC6926264

[CR18] Folstein, M. F., Folstein, S. E., & McHugh, P. R. (1975). *Mini-Mental State Examination (MMS, MMSE)* [Database record]. *APA PsycTests*. 10.1037/t07757-000

[CR19] Forbes, L., Gutierrez, D., & Johnson, S. K. (2018). Investigating adherence to an online introductory mindfulness program. *Mindfulness,**9*, 271–282. 10.1007/s12671-017-0772-4

[CR20] Fourteau, M., Virecoulon Giudici, K., Rolland, Y., Vellas, B., & de Souto Barreto, P. (2020). Associations between multidomain lifestyle interventions and intrinsic capacity domains during aging: a narrative review. *Journal of Aging Research and Lifestyle*, *9*, 16–25. 10.14283/jarlife.2020.6 10.14283/jarlife.2020.6 PMC10002877 36922921

[CR21] Geurts, D. E., Haegens, N. M., Van Beek, M. H., Schroevers, M. J., Compen, F. R., & Speckens, A. E. (2021). Putting mindfulness-based cognitive therapy to the test in routine clinical practice: A transdiagnostic panacea or a disorder specific intervention? *Journal of Psychiatric Research,**142*, 144–152. 10.1016/j.jpsychires.2021.07.043 34352560 10.1016/j.jpsychires.2021.07.043

[CR22] Goodall, K., Trejnowska, A., & Darling, S. (2012). The relationship between dispositional mindfulness, attachment security and emotion regulation. *Personality and Individual Differences,**52*(5), 622–626. 10.1016/j.paid.2011.12.008

[CR23] Gratz, K. L., & Roemer, L. (2004). Multidimensional assessment of emotion regulation and dysregulation: Development, factor structure, and initial validation of the difficulties in emotion regulation scale. *Journal of Psychopathology and Behavioral Assessment,**26*, 41–54.

[CR24] Gutierrez, D., Forbes, L., & Johnson, S. K. (2020). Physical and psychological health predict adherence to an online mindfulness program for college students. *Counseling and Values,**65*(2), 206–221. 10.1002/cvj.12138

[CR25] Han, A. (2022). Mindfulness-based interventions for older adults with dementia or mild cognitive impairment: A meta-analysis. *Clinical Gerontologist,**45*(4), 763–776. 10.1080/07317115.2021.1995561 34693892 10.1080/07317115.2021.1995561

[CR26] Hallion, L. S., Steinman, S. A., Tolin, D. F., & Diefenbach, G. J. (2018). Psychometric properties of the Difficulties in Emotion Regulation Scale (DERS) and its short forms in adults with emotional disorders. *Frontiers in Psychology,**9*, 539. 10.3389/fpsyg.2018.0053 29725312 10.3389/fpsyg.2018.00539 PMC5917244

[CR27] He, Z., Tian, S., Singh, A., Chakraborty, S., Zhang, S., Lustria, M. L. A., ... & Boot, W. R. (2022). A machine-learning based approach for predicting older adults’ adherence to technology-based cognitive training. *Information Processing & Management*, *59*(5), 103034. 10.1016/j.ipm.2022.103034 10.1016/j.ipm.2022.103034 PMC9337718 35909793

[CR28] Hearn, J. H., & Finlay, K. A. (2018). Internet-delivered mindfulness for people with depression and chronic pain following spinal cord injury: A randomized, controlled feasibility trial. *Spinal Cord,**56*(8), 750–761. 10.1038/s41393-018-0090-2 29581519 10.1038/s41393-018-0090-2

[CR29] Ibeggazene, S., Pymer, S., Birkett, S. T., Caldow, E., & Harwood, A. E. (2022). A systematic review of exercise intervention reporting quality and dose in studies of intermittent claudication. *Vascular,**31*(3), 477–488. 10.1177/17085381211070700 35130092 10.1177/17085381211070700 PMC10233510

[CR30] Joormann, J., & Stanton, C. H. (2016). Examining emotion regulation in depression: A review and future directions. *Behaviour Research and Therapy,**86*, 35–49. 10.1016/j.brat.2016.07.007 27492851 10.1016/j.brat.2016.07.007

[CR31] Kabat-Zinn, J. (1982). An outpatient program in behavioral medicine for chronic pain patients based on the practice of mindfulness meditation: Theoretical considerations and preliminary results. *General Hospital Psychiatry,**4*(1), 33–47. 10.1016/0163-8343(82)90026-3 7042457 10.1016/0163-8343(82)90026-3

[CR32] Krishnamoorthy, Y., Rajaa, S., & Rehman, T. (2020). Diagnostic accuracy of various forms of geriatric depression scale for screening of depression among older adults: Systematic review and meta-analysis. *Archives of Gerontology and Geriatrics,**87*, 104002. 10.1016/j.archger.2019.104002 31881393 10.1016/j.archger.2019.104002

[CR33] Lam, L. C. W., Chan, W. C., Leung, T., Fung, A. W. T., & Leung, E. M. F. (2015). Would older adults with mild cognitive impairment adhere to and benefit from a structured lifestyle activity intervention to enhance cognition?: A cluster randomized controlled trial. *PLoS ONE,**10*(3), e0118173. 10.1007/s11136-011-9954-z 25826620 10.1371/journal.pone.0118173 PMC4380493

[CR34] Liland, K,, Mevik, B., Wehrens, R. (2022). *pls: Partial Least Squares and Principal Component Regression*. R package version 2.8–1, https://CRAN.R-project.org/package=pls

[CR35] Mascaro, J. S., Wehrmeyer, K., Mahathre, V., & Darcher, A. (2020). A longitudinal, randomized and controlled study of app-delivered mindfulness in the workplace. *Journal of Wellness,**2*(1), 4. 10.18297/jwellness/vol2/iss1/4

[CR36] Mehmood, T., Liland, K. H., Snipen, L., & Sæbø, S. (2012). A review of variable selection methods in partial least squares regression. *Chemometrics and Intelligent Laboratory Systems,**118*, 62–69. 10.1016/j.chemolab.2012.07.010

[CR37] Mirabito, G., & Verhaeghen, P. (2023). The Effects of Mindfulness Interventions on Older Adults’ Cognition: A Meta-Analysis. *The Journals of Gerontology: Series B,**78*(3), 394–408. 10.1093/geronb/gbac143 10.1093/geronb/gbac143 36148552

[CR38] Morris, T. P., Burzynska, A., Voss, M., Fanning, J., Salerno, E. A., Prakash, R., ... & Kramer, A. F. (2021). Brain structure and function predict adherence to an exercise intervention in older adults. *Medicine and Science in Sports and Exercise*, *54*(9), 1483–1492. 10.1249/MSS.0000000000002949 10.1249/MSS.0000000000002949 PMC9378462 35482769

[CR39] Mrazek, M. D., Franklin, M. S., Phillips, D. T., Baird, B., & Schooler, J. W. (2013). Mindfulness training improves working memory capacity and GRE performance while reducing mind wandering. *Psychological Science,**24*(5), 776–781. 10.1177/0956797612459659 23538911 10.1177/0956797612459659

[CR40] Ngandu, T., Lehtisalo, J., Korkki, S., Solomon, A., Coley, N., Antikainen, R., ... & Kivipelto, M. (2022). The effect of adherence on cognition in a multidomain lifestyle intervention (FINGER). *Alzheimer's & Dementia*, *18*(7), 1325–1334. 10.1002/alz.12492 10.1002/alz.12492 34668644

[CR41] Park, D. C., & Reuter-Lorenz, P. (2009). The adaptive brain: Aging and neurocognitive scaffolding. *Annual Review of Psychology,**60*, 173–196. 10.1146/annurev.psych.59.103006.093656 19035823 10.1146/annurev.psych.59.103006.093656 PMC3359129

[CR42] Parsons, C. E., Crane, C., Parsons, L. J., Fjorback, L. O., & Kuyken, W. (2017). Home practice in Mindfulness-Based Cognitive Therapy and Mindfulness-Based Stress Reduction: A systematic review and meta-analysis of participants’ mindfulness practice and its association with outcomes. *Behaviour Research and Therapy,**95*, 29–41. 10.1016/j.brat.2017.05.004 28527330 10.1016/j.brat.2017.05.004 PMC5501725

[CR43] Picorelli, A. M. A., Pereira, L. S. M., Pereira, D. S., Felício, D., & Sherrington, C. (2014). Adherence to exercise programs for older people is influenced by program characteristics and personal factors: A systematic review. *Journal of Physiotherapy,**60*(3), 151–156. 10.1016/j.jphys.2014.06.012 25092418 10.1016/j.jphys.2014.06.012

[CR44] Prakash, R. S., Fountain-Zaragoza, S., Fisher, M., Gbadeyan, O., Andridge, R., Kiecolt-Glaser, J., ... & Canter, R. (2022). Protocol for a randomized controlled trial of mindfulness-based stress reduction to improve attentional control in older adults (HealthyAgers trial). *BMC geriatrics*, *22*(1), 666. 10.1186/s12877-022-03334-7 10.1186/s12877-022-03334-7 PMC9375078 35964000

[CR45] Reangsing, C., Punsuwun, S., & Schneider, J. K. (2021). Effects of mindfulness interventions on depressive symptoms in adolescents: A meta-analysis. *International Journal of Nursing Studies,**115*, 103848. 10.1016/j.ijnurstu.2020.103848 33383273 10.1016/j.ijnurstu.2020.103848

[CR46] Rush, A. J., Giles, D. E., Schlesser, M. A., Fulton, C. L., Weissenburger, J., & Burns, C. (1986). The inventory for depressive symptomatology (IDS): Preliminary findings. *Psychiatry Research,**18*(1), 65–87. 10.1016/0165-1781(86)90060-0 3737788 10.1016/0165-1781(86)90060-0

[CR47] Salthouse, T. A. (2010). Selective review of cognitive aging. *Journal of the International Neuropsychological Society,**16*(5), 754–760. 10.1017/S1355617710000706 20673381 10.1017/S1355617710000706 PMC3637655

[CR48] Samimy, S., Manglani, H. R., Fountain-Zaragoza, S., Andridge, R., & Prakash, R. S. (2022). Impact of mindfulness training on in-the-moment attentional control and emotion dysregulation in older adults: Secondary analysis of a pilot, placebo-controlled randomized controlled trial. *Aging & Mental Health,**26*(12), 2372–2380. 10.1080/13607863.2021.1998348 34894884 10.1080/13607863.2021.1998348

[CR49] Sanchez-Lara, E., Lozano-Ruiz, A., Perez-Garcia, M., & Caracuel, A. (2022). Efficacy of mindfulness-based interventions in cognitive function in the elderly people: A systematic review and meta-analysis. *Aging & Mental Health,**26*(9), 1699–1709. 10.1080/13607863.2021.1976724 34587844 10.1080/13607863.2021.1976724

[CR50] Sardella, A., Lenzo, V., Basile, G., Martino, G., & Quattropani, M. C. (2023). Emotion regulation strategies and difficulties in older adults: A systematic review. *Clinical Gerontologist,**46*(3), 280–301. 10.1080/07317115.2022.2128706 36163629 10.1080/07317115.2022.2128706

[CR51] Sawatsky, M. L., Clyde, M., & Meek, F. (2015). Partial least squares regression in the social sciences. *The Quantitative Methods for Psychology,**11*(2), 52–62. 10.20982/tqmp.11.2.p052

[CR52] Siebenhüner, A. R., Mikolasek, M., Witt, C. M., & Barth, J. (2021). Improvements in health might contradict adherence to mobile health interventions: findings from a self-care cancer app study. *Journal of Alternative and Complementary Medicine*, *27*(S1), S-115. 10.1089/acm.2020.0111 10.1089/acm.2020.0111 33788602

[CR53] Stanic, J., Barth, J., Danon, N., Bondolfi, G., Jermann, F., & Eicher, M. (2021). Adherence to standardized 8-week mindfulness-based interventions among women with breast or gynecological cancer: A scoping review. *Journal of Psychosocial Oncology Research and Practice,**3*(2), e048. 10.1097/OR9.0000000000000048

[CR54] Tamagawa, R., Speca, M., Stephen, J., Pickering, B., Lawlor-Savage, L., & Carlson, L. E. (2015). Predictors and effects of class attendance and home practice of yoga and meditation among breast cancer survivors in a mindfulness-based cancer recovery (MBCR) program. *Mindfulness,**6*, 1201–1210. 10.1007/s12671-014-0381-4

[CR55] Thompson, E. R. (2007). Development and validation of an internationally reliable short-form of the positive and negative affect schedule (PANAS). *Journal of Cross-Cultural Psychology,**38*(2), 227–242. 10.1177/0022022106297301

[CR56] Turunen, M., Hokkanen, L., Bäckman, L., Stigsdotter-Neely, A., Hänninen, T., Paajanen, T., ... & Ngandu, T. (2019). Computer-based cognitive training for older adults: Determinants of adherence. *PloS one*, *14*(7), e0219541.10.1371/journal.pone.0219541 10.1371/journal.pone.0219541 PMC6620011 31291337

[CR57] Vespa, J., Armstrong, D. M., & Medina, L. (2018). *Demographic turning points for the United States: Population projections for 2020 to 2060* (pp. 25–1144). Washington, DC: US Department of Commerce, Economics and Statistics Administration, US Census Bureau.

[CR58] Vujanovic, A. A., Bonn-Miller, M. O., Bernstein, A., McKee, L. G., & Zvolensky, M. J. (2010). Incremental validity of mindfulness skills in relation to emotional dysregulation among a young adult community sample. *Cognitive Behaviour Therapy,**39*(3), 203–213. 10.1080/16506070903441630 20182933 10.1080/16506070903441630 PMC2889232

[CR59] Wang, F. L., Tang, Q. Y., Zhang, L. L., Yang, J. J., Li, Y., Peng, H., & Wang, S. H. (2020). Effects of mindfulness-based interventions on dementia patients: A meta-analysis. *Western Journal of Nursing Research,**42*(12), 1163–1173. 10.1177/0193945920916750 32406791 10.1177/0193945920916750

[CR60] Wechsler, D. (2008). *Wechsler Adult Intelligence Scale-Fourth Edition (WAIS–IV)*. The Psychological Corporation.

[CR61] Weiss, N. H., Thomas, E. D., Schick, M. R., Reyes, M. E., & Contractor, A. A. (2022). Racial and ethnic differences in emotion regulation: A systematic review. *Journal of Clinical Psychology,**78*(5), 785–808. 34841522 10.1002/jclp.23284 PMC9035029

[CR62] Wong, C. G., Miller, J. B., Zhang, F., Rissman, R. A., Raman, R., Hall, J. R., ... & HABS-HD Study Team. (2023). Evaluation of Neighborhood-Level Disadvantage and Cognition in Mexican American and Non-Hispanic White Adults 50 Years and Older in the US. *JAMA Network Open*, 6(8), e2325325-e2325325 10.1001/jamanetworkopen.2023.25325 PMC10469291 37647071

[CR63] Winter, N., Russell, L., Ugalde, A., White, V., & Livingston, P. (2022). Engagement strategies to improve adherence and retention in web-based mindfulness programs: Systematic review. *Journal of Medical Internet Research,**24*(1), e30026. 10.2196/30026 35019851 10.2196/30026 PMC8792770

[CR64] Whitmoyer, P., Fountain-Zaragoza, S., Andridge, R., Bredemeier, K., Londeree, A., Kaye, L., & Prakash, R. S. (2020). Mindfulness training and attentional control in older adults: A randomized controlled trial. *Mindfulness,**11*, 203–218. 10.1007/s12671-019-01218-3

[CR65] Whitmoyer, P., Fisher, M. E., Duraney, E. J., Manzler, C., Isaacowitz, D. M., Andridge, R., & Prakash, R. S. (2023). Age differences in emotion regulation strategy use and flexibility in daily life. *Aging & Mental Health,* 1–14. 10.1080/13607863.2023.2256245 10.1080/13607863.2023.2256245 37735914

[CR66] Wu, C., Yi, Q., Zheng, X., Cui, S., Chen, B., Lu, L., & Tang, C. (2019). Effects of mind-body exercises on cognitive function in older adults: A meta-analysis. *Journal of the American Geriatrics Society,**67*(4), 749–758. 10.1111/jgs.15714 30565212 10.1111/jgs.15714

[CR67] Yesavage, J. A., Brink, T. L., Rose, T. L., Lum, O., Huang, V., Adey, M., & Leirer, V. O. (1982). Development and validation of a geriatric depression screening scale: A preliminary report. *Journal of Psychiatric Research,**17*(1), 37–49. 10.1016/0022-3956(82)90033-4 7183759 10.1016/0022-3956(82)90033-4

